# Optimal Pipette Resistance, Seal Resistance, and Zero-Current Membrane Potential for Loose Patch or Breakthrough Whole-Cell Recording *in vivo*

**DOI:** 10.3389/fncir.2020.00034

**Published:** 2020-06-30

**Authors:** Linqing Yan, Qi Fang, Xingui Zhang, Bowan Huang

**Affiliations:** ^1^Mental Health Center of Shantou University, Shantou, China; ^2^Department of Anesthesiology, Central People’s Hospital of Zhanjiang, Zhanjiang, China; ^3^Zilkha Neurogenetic Institute, Keck School of Medicine, University of Southern California, Los Angeles, CA, United States; ^4^College of Integrative Medicine, Fujian University of Traditional Chinese Medicine, Fuzhou, China

**Keywords:** *in vivo* recordings, pipette resistance, seal resistance, membrane potential, recording time

## Abstract

*In vivo* loose patch and breakthrough whole-cell recordings are useful tools for investigating the intrinsic and synaptic properties of neurons. However, the correlation among pipette resistance, seal condition, and recording time is not thoroughly clear. Presently, we investigated the recording time of different pipette resistances and seal conditions in loose patch and breakthrough whole-cell recordings. The recording time did not change with pipette resistance for loose patch recording (*R*_p_-loose) and first increased and then decreased as seal resistance for loose patch recording (*R*_s_-loose) increased. For a high probability of a recording time ≥30 min, the low and high cutoff values of *R*_s_-loose were 21.5 and 36 MΩ, respectively. For neurons with *R*_s_-loose values of 21.5–36 MΩ, the action potential (AP) amplitudes changed slightly 30 min after the seal. The recording time increased as seal resistance for whole-cell recording (*R*_s_-tight) increased and the zero-current membrane potential for breakthrough whole-cell recording (MP_zero-current_) decreased. For a high probability of a recording time ≥30 min, the cutoff values of *R*_s_-tight and MP_zero-current_ were 2.35 GΩ and −53.5 mV, respectively. The area under the curve (AUC) of the MP_zero-current_ receiver operating characteristic (ROC) curve was larger than that of the *R*_s_-tight ROC curve. For neurons with MP_zero-current_ values ≤ −53.5 mV, the inhibitory or excitatory postsynaptic current amplitudes did not show significant changes 30 min after the seal. In neurons with *R*_s_-tight values ≥2.35 GΩ, the recording time gradually increased and then decreased as the pipette resistance for whole-cell recording (*R*_p_-tight) increased. For the high probability of a recording time ≥30 min, the low and high cutoff values of *R*_p_-tight were 6.15 and 6.45 MΩ, respectively. Together, we concluded that the optimal *R*_s_-loose range is 21.5–36 MΩ, the optimal *R*_p_-tight range is 6.15–6.45 MΩ, and the optimal *R*_s_-tight and MP_zero-current_ values are ≥2.35 GΩ and ≤ −53.5 mV, respectively. Compared with *R*_s_-tight, the MP_zero-current_ value can more accurately discriminate recording times ≥30 min and <30 min.

## Introduction

*In vivo* loose patch recording and breakthrough whole-cell recording (hereafter, whole-cell recording) are important techniques in neuroscience (Sun et al., 2010; Zhou et al., 2010). However, the recording time is often short (Wang et al., 2016). The recording time is interrelated with the recording electrode’s condition (e.g., pipette resistance) and the recording electrode’s seal condition [e.g., seal resistance before rupturing the cell membrane or zero-current membrane potential (MP_zero-current_) without artificial interference after rupturing the cell membrane] (Neher et al., 1978; Hamill et al., 1981). The correlation among pipette resistance, seal condition, and recording time remains largely unclear. Solving this problem will help researchers to improve recording time, enhance their confidence in completing studies, and decrease training time.

*Via* loose patch recording, researchers can measure the suprathreshold firing of a single neuron and investigate the spiking properties of neurons (Tan et al., 2008; Sun et al., 2013). In loose patch recording, the pipette is usually sealed on the membrane patch that is invaginated into its lumen usually *via* suction (Roberts and Almers, 1992). The cell membrane is not ruptured (Neher et al., 1978). As the pipette resistance for loose patch recording (*R*_p_-loose) decreases, the tip diameter of the recording electrode increases. The larger patched area is harder to seal (Horn and Korn, 1992; Penner, 1995), and even if sealed, the seal is looser and easier to destroy (Hamill et al., 1981). In addition, a larger patch membrane fluctuates more easily and is ruptured by the strength (i.e., the negative pressure in the lumen of the recording electrode or resilience of the cell membrane), which invaginates the cell membrane into the lumen of the recording electrode (Roberts and Almers, 1992). As the *R*_p_-loose increases, the tip diameter of the recording electrode decreases. Thus, the cell membrane is easier to directly pierce by the tip of the recording electrode during recording because of the movement of the cell membrane (Wang et al., 2016). On one hand, when *R*_p_-loose is constant, the larger the seal resistance for loose patch recording (*R*_s_-loose) is, the larger the negative pressure in the lumen of the recording electrode or resilience of the cell membrane is (Roberts and Almers, 1992), and the easier it is to damage the cell membrane (Weiss et al., 1986; Milton and Caldwell, 1990; Roberts et al., 1990; Roberts and Almers, 1992). On the other hand, the smaller *R*_s_-loose is, the looser the seal is and the easier it is to destroy (Hamill et al., 1981). Taken together, *R*_p_-loose and *R*_s_-loose seem to correlate with recording time, but the details of their correlation need to be further investigated.

Using whole-cell recording, researchers can measure the subthreshold excitatory and inhibitory electrical activities of a neuron and explore the properties of specific ion channels or receptors and the effect of neuronal activity on the functionality of neural circuits (Wehr and Zador, 2003; Zhang et al., 2003; Wang et al., 2016). Moreover, during whole-cell recording, staining and pharmacological agents can be infused into the neuron to reveal neuronal morphology and carry out intracellular pharmacological studies (Wang et al., 2016). During whole-cell recording, as the cell membrane is invaginated into the lumen of the recording electrode, the recording electrode is attached to the neuron with a seal resistance for whole-cell recording (*R*_s_-tight) ≥ 1 GΩ, and subsequently, the cell membrane is ruptured (Horn and Brodwick, 1980; Hamill et al., 1981). As analyzed in the above loose patch recording description, a smaller pipette resistance for whole-cell recording (*R*_p_-tight) means a looser seal (Horn and Korn, 1992), which is easier to destroy (Hamill et al., 1981). A larger *R*_p_-tight means a smaller amount of patch membrane is involved, which makes fluctuations more difficult and increases the difficulty of rupture. The process of rupturing the membrane alters the mechanical stability of the seal with a higher probability. As the seal resistance increases (Hamill et al., 1981) and the zero-current membrane potential (MP_zero-current_) decreases (Hamill et al., 1981; Kornreich, 2007), the seal is mechanically more stable. Therefore, *R*_p_-tight, *R*_s_-tight, and MP_zero-current_ values all theoretically influence the recording time (Hamill et al., 1981). However, the detailed correlation among these factors remains unclear.

The *R*_p_-loose, *R*_p_-tight, and *R*_s_-loose values cannot be too small or too large. The *R*_s_-tight cannot be too small, and the MP_zero-current_ cannot be too high. Therefore, we hypothesize that for long-lasting recording, there are optimal ranges of *R*_p_-loose, *R*_s_-loose, and *R*_p_-tight. Additionally, there are optimal cutoff values for *R*_s_-tight and MP_zero-current_. To test our hypothesis in this study, we analyzed the data (*R*_p_-loose, *R*_s_-loose, *R*_p_-tight, *R*_s_-tight, MP_zero-current_, and recording time) of loose patch and whole-cell recordings in the primary auditory cortex and aimed to determine the correlation among pipette resistance, seal condition, and recording time.

## Methods

### Animal Preparation

All experimental procedures were approved by the Animal Care and Use Committee of Shantou University Medical College, Guangdong, China. C57BL/6J mice (female, 6–8 weeks, 16–20 g) with normal hearing were used in this study. The mice were housed under a 12-h light/dark cycle with water and food provided *ad libitum*. The mice were first anesthetized using sodium pentobarbital (60–70 mg/kg i.p., Sigma-Adrich, St. Louis, MO, USA), with an additional dose administered if the pedal withdrawal reflex was evoked by a toe pinch. The mouse body temperature was continuously monitored and maintained at 37°C using a heating pad with a feedback controller. Atropine sulfate (0.25 mg/kg, Nandao, Hainan, China) was injected subcutaneously to reduce secretions in the respiratory tracts. The heads of the mice were fixed using a customized apparatus with dental cement. Then, for approximately 3 days, the mice were then habituated, allowed to recover, and trained to be accustomed to head fixation on the recording setup.

### *In vivo* Recordings of Awake Mice

Before electrophysiological recordings, the mouse was anesthetized with isoflurane (2%; Sigma-Adrich, St. Louis, MO, USA), and a craniotomy was performed over the primary auditory cortex. The electrophysiological experiments, including loose patch recording and breakthrough whole-cell recording *in vivo*, were carried out on an anti-vibration table in a soundproof room, and the head of the mouse was immobilized as described above (Xiong et al., 2013).

After the mouse awakened from isoflurane anesthesia, the dura was removed, and a glass pipette (tip diameter of approximately 1.0 μm, Sutter, Inc., USA, vertically pulled by PC-10, Narishige, Tokyo, Japan) was inserted in the primary auditory cortex vertically to the brain surface controlled by a micromanipulator (Siskiyou Inc., Grants Pass, OR, USA). The pipette solution contained artificial cerebral spinal fluid (ACSF; in mM: 124 NaCl, 2.5 KCl, 2 CaCl_2_, 1 MgCl_2_, 25 NaHCO_3_, 1.23 NaH_2_PO_4_, 20 glucose, and 1.5% biocytin, pH 7.25, Sigma-Adrich, St. Louis, MO, USA) for loose patch recording or a synthetic fluid (in mM: 125 Cs-gluconate, 2 CsCl, 5 TEA-Cl, 4 MgATP, 0.3 GTP, 8 phosphocreatine, 10 HEPES, 10 EGTA, 1 QX-314, pH 7.23, Sigma-Adrich, St. Louis, MO, USA) for whole-cell recordings. After being inserted into an electrode holder (Axon Instruments), the patch pipette was first rapidly lowered to the desired depth (from L2/3 to L6, i.e., 151–1,000 μm) under 6-psi positive pressure; then, the pipette was slowly advanced (in 1 μm steps) at a lower pressure (0.5–1 psi) until a neuron was detected, which was reflected by a pipette resistance change. When the patch pipette obtained a low seal resistance (10–200 MΩ), the neuron recording was considered to be a loose patch recording. An Axoclamp 700B amplifier (Axon Instruments/Molecular Devices, Sunnyvale, CA, USA) was used in current-clamp mode for recording action potential (AP). When the patch pipette obtained a high seal resistance (≥1 GΩ) and the neuron membrane was successfully sucked through, the recording was considered to be a whole-cell recording that would allow the MP_zero-current_ to be evaluated in current-clamp mode (*I* = 0) first and then the recording mode could be switched to voltage-clamp mode to record the inhibitory postsynaptic current (IPSC) and excitatory postsynaptic current (EPSC). For recording the IPSC and EPSC, the membrane potential was held at 0 mV and −70 mV, respectively (Wu et al., 2011). For voltage-clamp recording, the whole cell and pipette were completely compensated for, and the initial series resistance (20–40 MΩ) was compensated for by 50–60% to achieve an effective series resistance of 10–20 MΩ (Wu et al., 2006). Electrical signals were filtered with a bandpass filter (300–3,000 Hz) and sampled at 20 kHz.

### Sound Calibration and Generation

White noise stimuli with various frequencies (2–45 kHz, 0.1-octave interval) were generated and delivered to the contralateral ear of mice using a Tucker-Davis Technologies System 3 (TDT 3, Tucker-Davis Technologies, Alachua, FL, USA). A real-time processor (RP 2.1) and a custom-made program written with RPvdsEx software were used to generate the sound signals, and the intensities of the sound were controlled by a programmable attenuator (PA5). The synthesized signals were amplified and delivered through an electrostatic speaker driver (ED1) and a free-field ultrasonic loudspeaker (ES1; frequency range, 2–110 kHz). The loudspeaker was calibrated with 1/8- and 1/4-inch microphones (Brüel and Kjaer 4138, 4135, Naerum, Denmark) and an amplifier (Brüel and Kjaer 2610, Naerum, Denmark) before the experiment. The noise parameters (60 dB SPL, 50-ms duration, 5-ms rise–fall time) were controlled by Brain Ware software. The noises were repeated until no recognizable APs, IPSCs, or EPSCs were observed and the interstimulus interval was 500 ms.

### Data Processing

The *R*_p_-loose, *R*_p_-tight, *R*_s_-loose, *R*_s_-tight, MP_zero-current_, and recording time were documented. To investigate the correlation among the above variables, scatterplots of the two above variables were plotted. The receiver operating characteristic (ROC) curve is a tool to find the optimal cutoff value of a variable to better predict a positive event in many studies (Søreide, 2009; Mandrekar, 2010). In this study, to find the optimal cut-off values of a variable for a high probability of recording time ≥30 min, the ROC curve was determined, and the area under the curve (AUC) and Youden index were computed (Mandrekar, 2010). For offline data processing, the APs from loose patch recording were extracted *via* a custom-made MATLAB program. The IPSCs and EPSCs from the whole-cell recording were directly extracted *via* Clampex 12 (Axon, USA). Then, the amplitudes of APs, IPSCs, and EPSCs at 5 and 30 min were measured and compared.

All statistical analyses were performed with SPSS statistical software (version 13). The measurement data were presented as the mean ± SE and were first tested for normal distribution (Shapiro–Wilk test) and equal variances (Levene’s test) before performing appropriate parametric tests. For two-group comparisons, a two-tailed unpaired *t*-test (for normally distributed data) or Mann–Whitney *U* test (for nonnormally distributed data) was applied to evaluate significance. The enumeration data were tested with the Chi-square test. A *p*-value of less than 0.05 was considered to indicate significance.

## Results

### Correlation Among *R*_p_-loose, *R*_s_-loose, and Recording Time in Loose Patch Recording

**Figure 1:**
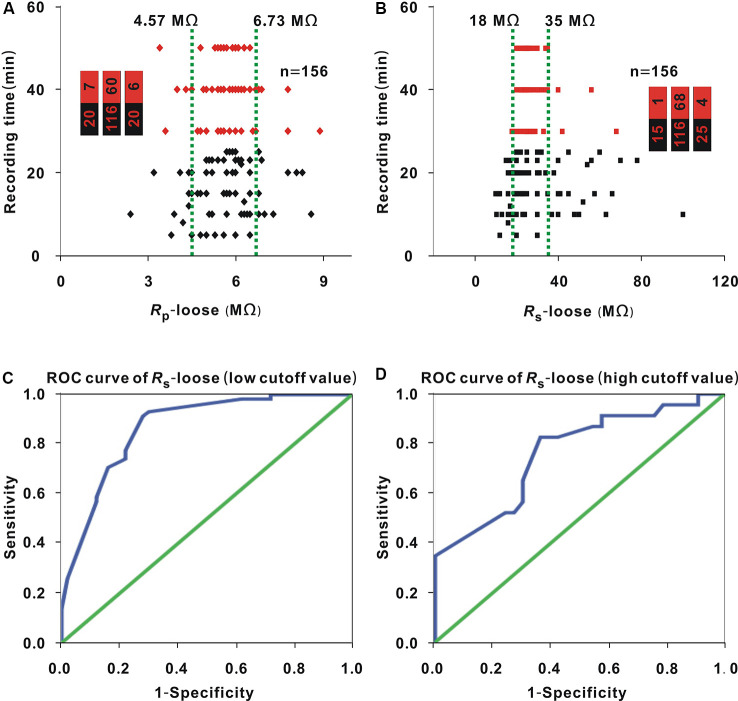
Influence of *R*_p_-loose and *R*_s_-loose on recording time. **(A)** Change in recording time with *R*_p_-loose (Chi-square test, *P* > 0.05). Red dots represent neurons (≥30 min). The two green lines were the cut off values to divide the entire interval of the abscissa variable into three small intervals, and the numbers in the bar chart were the number of neurons (≥30 min)/total number in the three small intervals (corresponding to values from left to right); panel **(B)** is depicted in a similar manner. **(B)** Change in recording time with *R*_s_-loose (Chi-square test, *P* < 0.01). Red dots represent neurons (≥30 min). **(C)** Receiver operating characteristic (ROC) curve to find the low cutoff value of *R*_s_-loose for a high probability of a recording time ≥30 min area under the curve [area under the curve (AUC) = 0.87, and low cutoff value = 21.5 MΩ]. **(D)** ROC curve to find the high cut off value of* R*_s_-loose for a high probability of a recording time ≥30 min (AUC = 0.76, and high cutoff value = 36 MΩ).

Loose patch recording was carried out in 156 neurons. *R*_p_-loose ranged from 2.4 to 8.9 MΩ, *R*_s_-loose ranged from 10 to 100 MΩ, and the recording time ranged from 5 to 50 min. As *R*_p_-loose increased, the recording time did not seem to be significantly changed (Figure 1A). The entire interval of *R*_p_-loose was averagely divided into three small intervals [the range of any small interval was (biggest *R*_p_-loose − smallest *R*_p_-loose)/3] (Figure 1A, two green lines). The proportion of neurons with a recording time ≥30 min (hereafter, neurons ≥30 min; Figure 1A, red dots) did not present significant change with *R*_p_-loose values in three small intervals (Chi-square test, *χ*^2^ = 4.52, *P* = 0.11). The *R*_s_-loose values of neurons (≥30 min; Figure 1B, red dots) were mostly in the range of 18–35 MΩ. When 18 and 35 MΩ were used as the cutoff values, the proportion of neurons (≥30 min; Figure 1B, red dots) first increased and then decreased as *R_s_-loose* increased (Chi-square test, *χ*^2^ = 25.74, *P* = 2.58 × 10^−6^).

**Figure 2:**
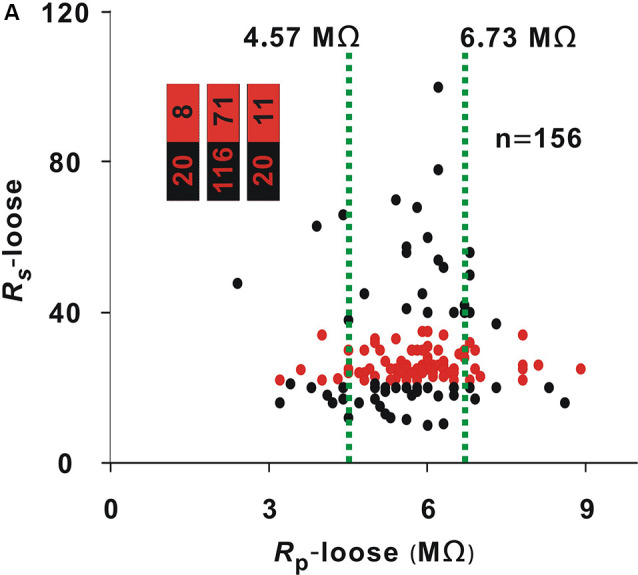
Influence of *R*_p_-loose on *R*_s_-loose. Change in *R*_s_-loose with *R*_p_-loose (Chi-square test, *P* > 0.05). Red dots represent neurons (21.5–36 MΩ). The two green lines were the cut off values to divide the entire interval of the abscissa variable into three small intervals; numbers in the bar chart were the number of neurons (21.5–36 MΩ)/total number in the three small intervals (corresponding to values from left to right).

A recording time ≥30 min was considered a successful recording. The optimal interval of *R*_s_-loose should include two cutoff values. To find the two cutoff values, we first determined the *R*_s_-loose values with the highest proportion of neurons (≥30 min). With a 3-MΩ step, the entire interval of 18–35 MΩ could be divided into six small intervals. The number of neurons (≥30 min)/(total number) in these small intervals of 18–20 MΩ, 21–23 MΩ, 24–26 MΩ, 27–29 MΩ, 30–32 MΩ, and 33–35 MΩ were 3/24, 17/20, 33/41, 8/8, 5/15, and 2/8, respectively. In the small interval of 27–29 MΩ, the proportion of neurons (≥30 min) was highest (8/8). To find the low cutoff value, the data of neurons with a *R*_s_-loose value ≤ 29 MΩ were used to draw the ROC curve (Figure 1C), and the AUC was 0.87. According to the Youden index, the low cutoff value was 21.5 MΩ. Similarly, the data of neurons with a *R*_s_-loose value ≥ 27 MΩ were used to draw the ROC curve to find the high cutoff value (Figure 1D), and the AUC was 0.76. According to the Youden index, the high cutoff value was 36 MΩ. Using a similar method to that used in Figure 1A, the proportion of neurons with a *R*_s_-loose value of 21.5–36 MΩ [hereafter, neurons (21.5–36 MΩ); Figure 2, red dots] was not influenced by *R*_p_-loose (Chi-square test, *χ*^2^ = 3.21, *P* = 0.20).

### Recording Quality of Neurons With a *R*_s_-loose Value of 21.5–36 MΩ and a Recording Time ≥30 min in Loose Patch Recording

**Figure 3:**
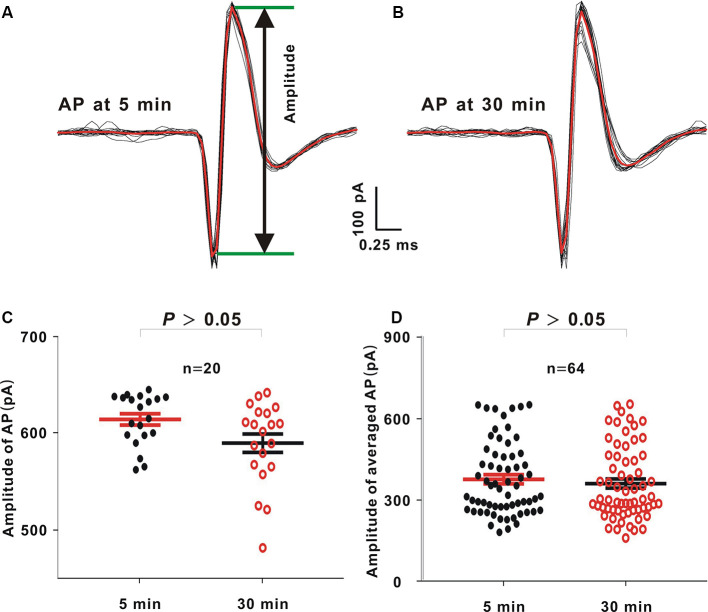
Comparison between action potential (AP) amplitudes at 5 and 30 min after sealing in loose patch recording. **(A,B)** In an example neuron, 60 dB noise evoked APs 5 and 30 min after sealing. The averaged APs (red lines) were obtained by averaging 20 APs (black lines) to the same acoustic stimulus. **(C)** AP amplitudes from the example neuron at 5 and 30 min (mean = 613.45 and *SE* = 5.88 at 5 min, and mean = 588.99 and *SE* = 8.52 at 30 min, Mann–Whitney *U* test, *P* > 0.05). **(D)** Averaged AP amplitudes from all neurons at 5 and 30 min (mean = 377.79 and *SE* = 16.79 at 5 min, and mean = 362.10 and *SE* = 16.78 at 30 min, Mann–Whitney *U* test, *P* > 0.05).

In this study, there were 90 neurons (21.5–36 MΩ). Among these 90 neurons, there were 64 neurons (≥30 min; 71.11%). In an example neuron, 60 dB noise stimulation with 20 repetitions was delivered, and we recorded APs 5 and 30 min after sealing (Figures 3A,B, black lines). The AP waveforms were then averaged (Figures 3A,B, red lines). The voltage difference from the positive peak to the negative valley was defined as the AP amplitude (Figure 3A, the voltage between two green lines). The amplitudes of 20 APs or 64 averaged APs at 5 min were similar to those at 30 min in the example neuron (Figure 3C, Mann–Whitney *U* test, *Z* = −1.89, *P* = 0.06) or in all recorded neurons (Figure 3D, Mann–Whitney *U* test, *Z* = −0.74, *P* = 0.45).

### Correlation Among *R*_p_-Tight, *R*_s_-Tight, MP_zero-current_, and Recording Time in Whole-Cell Recording

**Figure 4:**
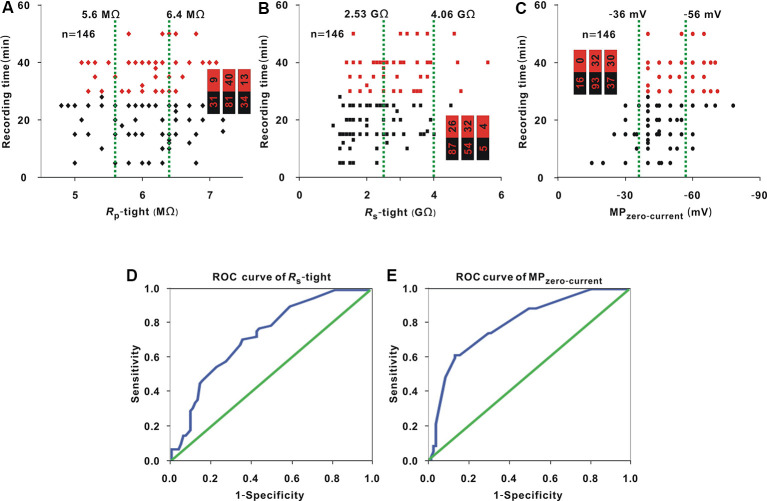
Influence of *R*_p_-tight, *R*_s_-tight, and MP_zero-current_ on recording time. **(A)** Change in recording time with *R*_p_-tight (Chi-square test, *P* > 0.05). Red dots represent neurons (≥30 min), which is the same in panels **(B,C)**. The two green lines are the cutoff values to divide the entire interval of the abscissa variable into three small intervals; the numbers in the bar chart are the number of neurons (≥30 min)/total number in the three small intervals (corresponding to values from left to right); the same is the case in panels **(B,C)**. **(B)** Change in recording time with *R*_s_-tight (Chi-square test, *P* < 0.01). **(C)** Change in recording time with MP_zero-current_ (Chi-square test, *P* < 0.01). **(D)** ROC curve to find the cutoff value of *R*_s_-tight for a high probability of a recording time ≥30 min (AUC = 0.73, cutoff value = 2.35 GΩ). **(E)** ROC curve to find the cutoff value of MP_zero-current_ for a high probability of a recording time ≥30 min (AUC = 0.80, cutoff value = −53.5 mV).

Whole-cell recording was carried out in 146 neurons. *R*_p_-tight ranged from 4.8 to 7.2 MΩ; *R*_s_-tight ranged from 1 to 5.6 GΩ; MP_zero-current_ ranged from −78 to −15 mV; and the recording time ranged from 5 to 50 min. With the same method as that used in Figure 1A, in three small intervals, the proportion of neurons (≥30 min; Figure 4A, red dots) did not significantly change as *R*_p_-tight increased (Chi-square test, *χ*^2^ = 4.13, *P* = 0.13), the proportions of neurons (≥30 min; Figures 4B,C, red dots) gradually increased as *R*_s_-tight increased (Chi-square test, *χ*^2^ = 14.75, *P* = 0.63 × 10^−3^), and MP_zero-current_ decreased (Chi-square test, *χ*^2^ = 36.86, *P* = 9.90 × 10^−9^). A recording time ≥30 min was defined as a successful recording. To find the cutoff values of *R*_s_-tight and MP_zero-current_ for a high probability of a recording time ≥30 min, the ROC curves of *R*_s_-tight and MP_zero-current_ were plotted (Figures 4D,E). The AUC of *R*_s_-tight was 0.73, which was smaller than that of MP_zero-current_ (0.80). According to the Youden index, the cutoff value of *R*_s_-tight was 2.35 GΩ, and the cutoff value of MP_zero-current_ was −53.5 mV.

**Figure 5:**
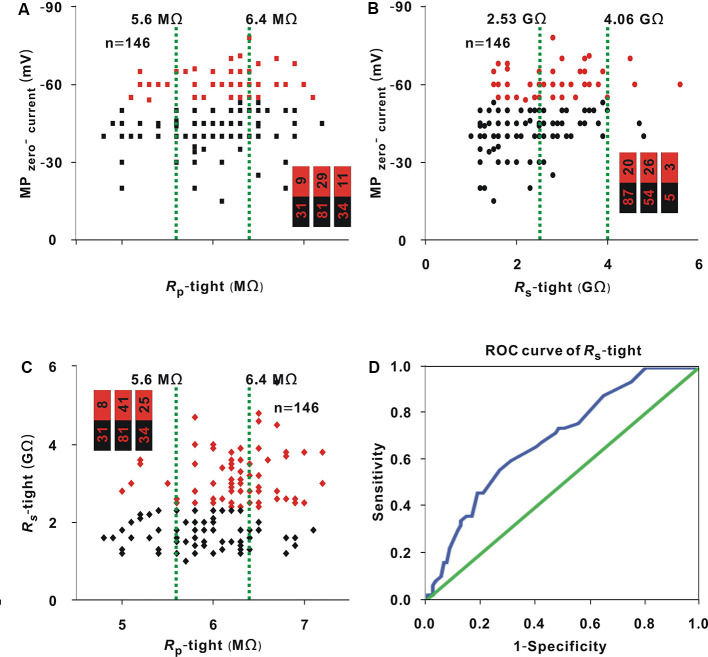
Correlation among MP_zero-current_, *R*_s_-tight, and *R*_p_-tight. **(A)** Change in MP_zero-current_ with *R*_p_-tight (Chi-square test, *P* > 0.05). Red dots represent neurons (−53.5 mV). The two green lines are the cutoff values to divide the entire interval of the abscissa variable into three small intervals, and the numbers in the bar chart were the number of neurons (−53.5 mV)/total number in the three small intervals (corresponding to values from left to right); this is also the case in panels **(B,C)**. **(B)** Change in MP_zero-current_ with *R*_s_-tight (Chi-square test, *P* < 0.01). Red dots represent neurons (≤ −53.5 mV). **(C)** Changes in *R*_s_-tight with *R*_p_-tight (Chi-square test, *P* < 0.01). Red dots represent neurons (≥2.35 GΩ). **(D)** ROC curve to find the cutoff value of* R*_s_-tight for a high probability of a MP_zero-current_ ≤ −53.5 mV (AUC = 0.69, cutoff value = 2.70 GΩ).

As shown in the analysis in Figure 1A, in the three small intervals, the proportion of neurons with MP_zero-current_ values ≤ −53.5 mV [hereafter, neurons (≤ −53.5 mV; Figure 5A, red dots)] did not change with *R*_p_-tight (Chi-square test, *χ*^2^ = 0.49, *P* = 0.78), and the proportion of neurons (≤ −53.5 mV) or neurons with a *R*_s_-tight value ≥ 2.35 GΩ [hereafter, neurons (≥2.35 GΩ; Figures 5B,C, red dots)] gradually increased as *R*_s_-tight (Chi-square test, *χ*^2^ = 11.08, *P* = 3.92 × 10^−3^) or *R*_p_-tight (Chi-square test, *χ*^2^ = 14.78, *P* = 0.62 × 10^−3^) increased. A MP_zero-current_ value ≤ −53.5 mV was defined as a successful seal. To find the cutoff value of *R*_s_-tight for the high probability of a MP_zero-current_ value ≤ −53.5 mV, the ROC curve of *R*_s_-tight was plotted (Figure 5D). The AUC was 0.69. According to the Youden index, the cutoff value of *R*_s_-tight was 2.70 GΩ, which was similar to the cutoff value (2.35 GΩ) of *R*_s_-tight for a high probability of a recording time ≥30 min.

### Recording Quality of Neurons With a MP_zero-current_ Value ≤ −53.5 mV and a Recording Time ≥30 min in Whole-Cell Recording

**Figure 6:**
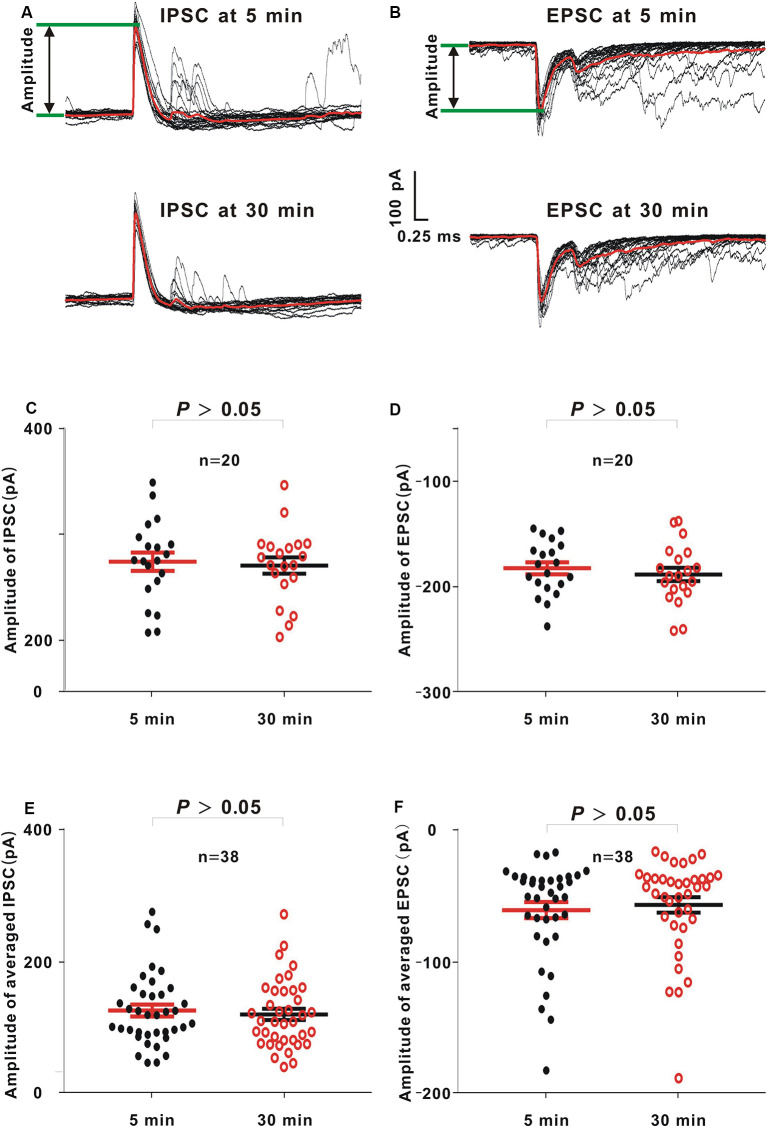
Comparison between amplitudes of inhibitory postsynaptic currents (IPSCs) or excitatory postsynaptic currents (EPSCs) 5 and 30 min after sealing in whole-cell recording. **(A,B)** In an example neuron, 60-dB noise-evoked IPSCs **(A)** or EPSCs **(B)** 5 and 30 min after the seal. The averaged IPSCs or EPSCs (red lines) were obtained by averaging 20 IPSCs or EPSCs (black lines) to the same acoustic stimulus. **(C,D)** IPSC or EPSC amplitudes from the example neuron at 5 and 30 min (IPSC, mean = 274.47 and *SE* = 8.74 at 5 min, and mean = 270.75 and *SE* = 7.79 at 30 min, unpaired *t* test, *P* > 0.05; EPSC, mean = −182.29 and *SE* = 5.72 at 5 min, and mean = −188.14 and *SE* = 6.34 at 30 min, Mann–Whitney *U* test, *P* > 0.05). **(E,F)** Averaged IPSC or EPSC amplitudes from all neurons at 5 and 30 min (IPSC, mean = 125.18 and *SE* = 8.89 at 5 min, and mean = 119.31 and *SE* = 8.41 at 30 min, unpaired *t*-test, *P* > 0.05; EPSC, mean = −61.02 and *SE* = 6.07 at 5 min, and mean = −57.05 and *SE* = 5.81 at 30 min, Mann–Whitney *U* test, *P* > 0.05).

In this study, there were 49 neurons (≤ −53.5 mV), among which there were 38 neurons (≥30 min; 77.55%). Similar to the loose patch recording approach, 60-dB noise stimulation with 20 repetitions was presented to evoke IPSCs and EPSCs (Figures 6A,B, black lines, an example neuron). Then, the averaged IPSC and EPSC were obtained (Figures 6A,B, red line). The IPSC and EPSC amplitudes were extracted by measuring the voltage difference from the baseline to the positive or negative peak (Figures 6A,B, the voltage between two green lines). In the example neuron, the amplitudes of 20 IPSCs or EPSCs at 5 min were not significantly different from those at 30 min (IPSC, unpaired *t*-test, *t* = 0.32, *P* = 0.75; Figure 6C; EPSC, Mann–Whitney *U* test, *Z* = −0.62, *P* = 0.53; Figure 6D). Moreover, when the averaged IPSC or EPSC was used to measure the amplitude, in 38 recorded neurons, the amplitudes of averaged IPSC or EPSC at 5 min were consistent with those at 30 min (IPSC, unpaired *t*-test, *t* = 0.48, *P* = 0.63; Figure 6E; EPSC, Mann–Whitney *U* test, *Z* = −0.46, *P* = 0.64; Figure 6F).

### Correlation Between *R*_p_-Tight and MP_zero-current_ or Recording Time in Neurons (≥2.35 GΩ) or Neurons With a *R*_s_-Tight <2.35 GΩ in Whole-Cell Recording

In whole-cell recording, the MP_zero-current_ and recording time were associated with *R*_s_-tight (Figures 4B, 5B), and *R*_s_-tight was associated with *R*_p_-tight (Figure 5C). Thus, the MP_zero-current_ and recording time should theoretically depend on *R*_p_-tight. However, this dependence is not supported by our results (Figures 4A, 5A). To further examine the correlation between *R*_p_-tight value and MP_zero-current_ value or recording time, we divided the neurons in whole-cell recording into two groups, neurons (≥2.35 GΩ) and neurons with a *R*_s_-tight < 2.35 GΩ [hereafter, neurons (<2.35 GΩ)].

**Figure 7:**
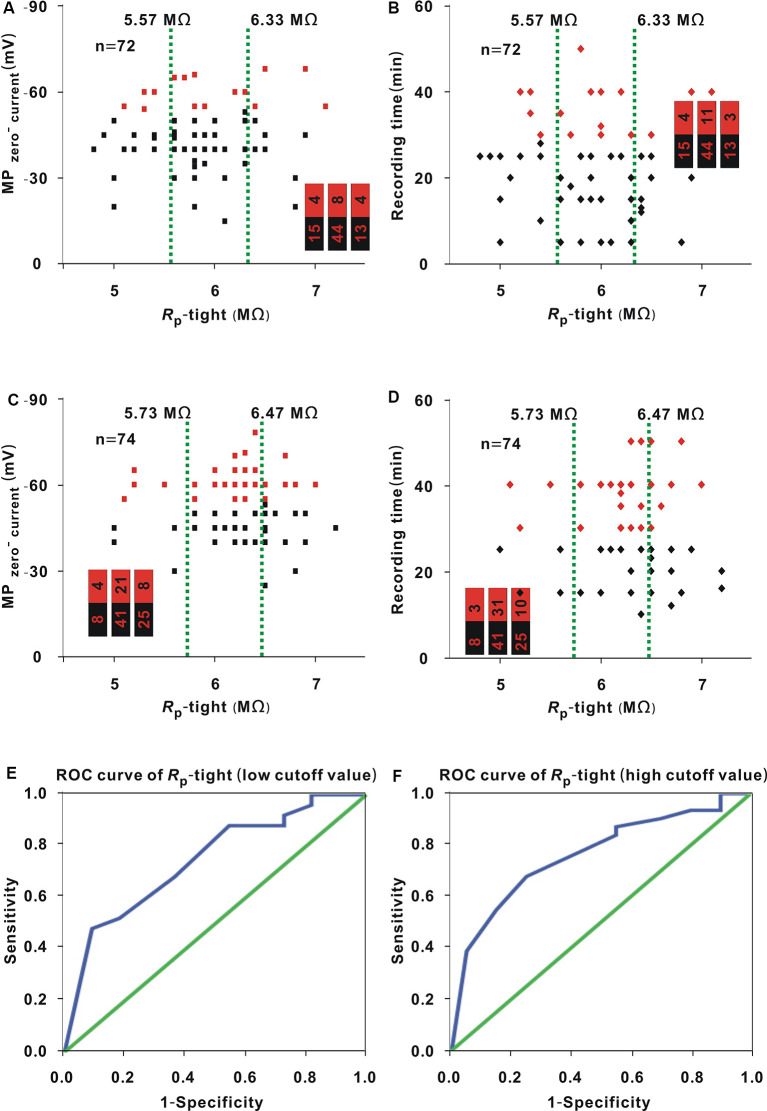
Influence of *R*_p_-tight on the MP_zero-current_ and recording time in neurons (≥2.35 GΩ) or neurons (<2.35 GΩ) for whole-cell recording. **(A)** Change in MP_zero-current_ with *R*_p_-tight in neurons (<2.35 GΩ; Chi-square test, *P* > 0.05). Red dots represent neurons (≤ −53.5 mV). The two green lines were the cutoff values to divide the entire interval of the abscissa variable into three small intervals, and the numbers in the bar chart were the number of neurons (≤ −53.5 mV)/total number in the three small intervals (corresponding to values from left to right); this is also the case in panels **(B–D)**. **(B)** Change in recording time with *R*_p_-tight in neurons (<2.35 GΩ; Chi-square test, *P* > 0.01). Red dots represent neurons (≥30 min). **(C)** Change in MP_zero-current_ with *R*_p_-tight in neurons (≥2.35 GΩ; Chi-square test, *P* > 0.05). Red dots represent neurons (≤ −53.5 mV). **(D)** Changes in recording time with *R*_p_-tight in neurons (≥2.35 GΩ; Chi-square test, *P* < 0.01). Red dots represent neurons (≥30 min). **(E)** ROC curve to find the low cutoff value of *R*_p_-tight for a high probability of a recording time ≥30 min in neurons (≥2.35 GΩ; AUC = 0.75, low cutoff value = 6.15 MΩ). **(F)** ROC curve to find the high cutoff value of *R*_p_-tight for a high probability of a recording time ≥30 min in neurons (≥2.35 GΩ; AUC = 0.76, high cutoff value = 6.45 MΩ).

The data in Figures 7A–D were analyzed with similar methods to that used in Figure 1A. In neurons (<2.35 GΩ), the proportion of neurons (≤ −53.5 mV; Figure 7A, red dots) or neurons (≥30 min; Figure 7B, red dots) did not show a significant change with *R*_p_-tight (MP_zero-current_: Chi-square test, *χ*^2^ = 1.14, *P* = 0.57; recording time: Chi-square test, *χ*^2^ = 0.05, *P* = 0.98). In neurons (≥2.35 GΩ), the proportion of neurons (≤ −53.5 mV; Figure 7C, red dots) exhibited no significant change with *R*_p_-tight (Chi-square test, *χ*^2^ = 2.43, *P* = 0.30); the proportion of neurons (≥30 min; Figure 7D, red dots) first showed an increase and then a decrease as *R*_p_-tight increased (Chi-square test, *χ*^2^ = 9.96, *P* = 6.86 × 10^−3^).

In the second small interval of 5.73–6.47 MΩ (Figure 7D), we further examined the seven small intervals determined in the 1-MΩ step. The numbers of neurons (≥30 min)/(total number) in the small intervals of 5.8 MΩ, 5.9 MΩ, 6.0 MΩ, 6.1 MΩ, 6.2 MΩ, 6.3 MΩ, and 6.4 MΩ were 5/7, 0/0, 4/6, 1/2, 12/13, 5/7, and 4/6, respectively. In the small interval of 6.2 MΩ, the proportion of neurons (≥30 min) was highest (12/13). To find the low cutoff value, the data of neurons with a *R*_p_-tight ≤6.2 MΩ were used to plot the ROC curve (Figure 7E). The AUC was 0.75, and the low cutoff value was 6.15 MΩ based on the Youden index. Similarly, the data of neurons with a *R*_p_-tight ≥6.2 MΩ were used to plot the ROC curve and determine the high cutoff value (Figure 7F). The AUC was 0.76, and the high cutoff value was 6.45 MΩ from the Youden index.

## Discussion

### *R*_p_-loose and *R*_p_-Tight for a High Probability of a Recording Time ≥30 min

An AUC value ≥0.7 of the ROC suggests that the prediction performance of a variable is acceptable (Mandrekar, 2010). In neurons (≥2.35 GΩ), the AUC values of the two ROC curves for finding low and high cutoff values of *R*_p_-tight were larger than 0.7. Therefore, 6.15–6.45 MΩ were optimal *R*_p_-tight values for a high probability of a recording time ≥30 min, which is similar to the *R*_p_-tight values used in the report by Malboubi et al. (2009). This result supported the analysis in the “Introduction” section, which states that *R*_p_-tight cannot be too small or too large. However, in loose patch recording or neurons (<2.35 GΩ), the *R*_p_-loose or *R*_p_-tight did not influence the recording time (Figures 1A, 7B). This lack of influence may be because the *R*_p_-loose or *R*_p_-tight in our study may be in the optimal range for having a high probability of a recording time ≥30 min or because the seal is relatively loose in loose patch recording or neurons (<2.35 GΩ), and the recording time is greatly influenced by the mechanical stability of the seal, not by *R*_p_-loose or *R*_p_-tight value.

### *R*_s_-loose or *R*_s_-Tight for a High Probability of a Recording Time ≥30 min

The AUC values of ROC curves to find the cutoff values of *R*_s_-loose or *R*_s_-tight were larger than 0.7 (Mandrekar, 2010). Consequently, it was suitable that for a high probability of a recording time ≥30 min, the optimal *R*_s_-loose was 21.5–36 MΩ and the optimal *R*_s_-tight was ≥2.35 GΩ. In neurons (21.5–36 MΩ), 71.11% of neurons could be recorded ≥30 min; at 30 min after sealing, the AP amplitudes remained the same (Figures 3C,D). These results further support that *R*_s_-loose values of 21.5–36 MΩ are suitable. In theory, higher *R*_s_-loose or *R*_s_-tight value means that the mechanical stability of the seal is higher. However, loose patch recording is a form of extracellular recording without rupturing the membrane (Neher et al., 1978). Different from whole-cell recording, if *R*_s_-loose reaches GΩ, the AP is not usually recognizable in loose patch recording. Therefore, *R*_s_-loose needs to be smaller, and the electrode tip and cell membrane cannot be too clean in loose patch recording. Due to the uncleanliness, suction is usually used to reach the target *R*_s_-loose value (Roberts and Almers, 1992). The larger the strength of suction is, the higher *R*_s_-loose is, and the easier it is to damage the cell membrane or seal (Weiss et al., 1986; Milton and Caldwell, 1990; Roberts et al., 1990; Roberts and Almers, 1992). That is, different from *R*_s_-tight, as *R*_s_-loose further increases, the recording time conversely decreases.

After determining the optimal *R*_s_-loose value, we can adjust *R*_s_-loose to the target value *via* suction or advancing or retracting the recording electrode. Whole-cell recording requires higher *R*_s_-tight (≥1 GΩ; Hamill et al., 1981), which is very difficult to achieve by only suction, or altering the depth of the recording electrode. To achieve optimal *R*_s_-tight value, it is important to keep the electrode tip and cell membrane clean (Hamill et al., 1981; Stett et al., 2003; Kornreich, 2007) and the recording system steady (Wang et al., 2016).

### MP_zero-current_ for a High Probability of a Recording Time ≥30 min

In addition to *R*_s_-tight, the MP_zero-current_ value also reflects the seal state. A lower MP_zero-current_ value means a higher mechanical stability of the seal (Hamill et al., 1981; Kornreich, 2007). As the AUC value of the ROC curve increases, the prediction performance of a variable becomes better (Mandrekar, 2010). The AUC of the ROC curve to find the cutoff value of MP_zero-current_ (0.80) was larger than that of *R*_s_-tight (0.73). Therefore, compared with *R*_s_-tight, the MP_zero-current_ is a better index for predicting a high probability of a recording time ≥30 min. The optimal MP_zero-current_ value was ≤ −53.5 mV. This optimal value was also supported by a high proportion of neurons (≥30 min) and a high recording quality of IPSC and EPSC in neurons (≤ −53.5 mV; Figures 6E,F).

The MP_zero-current_ reflects the eventual seal state. For whole-cell recording, if the *R*_s_-tight value is ≥1 GΩ, the cell membrane needs to be artificially ruptured (Hamill et al., 1981). During the rupturing process, the stability of the seal may decrease. Therefore, *R*_s_-tight does not reflect the eventual seal state and results in worse prediction performance in comparison with the MP_zero-current_. Reaching the optimal *R*_s_-tight value does not mean that the optimal MP_zero-current_ can be achieved. It is still necessary for the experimenter to improve his or her skill regarding rupturing membrane to obtain the optimal MP_zero-current_.

### Correlation Between Seal Resistance and MP_zero-current_ or Pipette Resistance in Loose Patch or Whole-Cell Recording

Both *R*_s_-tight and MP_zero-current_ represent seal condition. *R*_s_-tight was related to the MP_zero-current_ (Figures 5B,D). For a high probability of an MP_zero-current_ ≤ −53.5 mV, the optimal *R*_s_-tight was ≥2.70 GΩ, which was similar to ≥2.35 GΩ for a high probability of a recording time ≥30 min. Notably, 2.35 GΩ was determined by directly analyzing the recording time and was smaller than 2.70 GΩ. We considered that the value ≥2.35 GΩ was optimal for *R*_s_-tight. *R*_s_-tight changed with *R*_p_-tight (Figure 5C). This result was different from that of *R*_s_-loose, which was not influenced by *R*_p_-loose (Figure 2A). This difference may also be attributed to the fact that *R*_p_-loose had been in the optimal range or that the seal is relatively loose in the loose patch recording, and *R*_s_-loose is mostly dependent on the mechanical stability of the seal, not on the *R_p_-loose*.

### Strengths and Limitations of This Study

In previous studies, *R*_p_-loose (Roberts and Almers, 1992), *R*_p_-tight (Hamill et al., 1981; Malboubi et al., 2009), *R*_s_-loose (Roberts and Almers, 1992), *R*_s_-tight (Neher et al., 1978; Malboubi et al., 2009), or MP_zero-current_ (Hamill et al., 1981) values vary greatly. In this study, for long-lasting *in vivo* loose patch or whole-cell recordings, we applied the ROC curve to analyze the recording time for obtaining optimal *R*_p_-loose, *R*_p_-tight, *R*_s_-loose, *R*_s_-tight, or MP_zero-current_ values. However, this study has some limitations. First, the recording time can be influenced by other factors (craniotomy quality (Lee et al., 2014), animal movement (Lee and Lee, 2017), brain pulsation (Levy et al., 2012), and cleanliness of the recording electrode (Hamill et al., 1981; Stett et al., 2003). These other factors cannot be controlled based on identical standards and may cause a biased result. Second, according to our experiment, a recording time ≥30 min was defined as a successful recording. When the cutoff value of the recording time was not 30 min, the results would be different. Third, the cell type and size can influence the selection of electrode or seal parameters (Penner, 1995). Our data were acquired from neurons in the primary auditory cortex, most of which have a size of at least 10 μm (Gopal and Gross, 1996; Hinova-Palova et al., 2018) and a MP_zero-current_ of −70 mV (Zhao et al., 2015). For smaller neurons (Tucker et al., 1979) or other cells (Lacampagne et al., 1996; Euler and Wässle, 1998), the optimal *R*_p_-loose, *R*_p_-tight, *R*_s_-loose, *R*_s_-tight, or MP_zero-current_ values may be different.

## Conclusions

For a high probability of a recording time ≥30 min, 21.5–36 MΩ is the optimal *R*_s_-loose value, 6.15–6.45 MΩ is the optimal *R*_p_-tight value, ≥2.35 GΩ is the optimal *R*_s_-tight value, and ≤ −53.5 mV is the optimal MP_zero-current_ value. Additionally, the MP_zero-current_ is better than *R*_s_-tight for predicting a positive event.

## Data Availability Statement

All datasets generated for this study are included in the article.

## Ethics Statement

The animal study was reviewed and approved by the Animal Care and Use Committee of Shantou University Medical College, Guangdong, China.

## Author Contributions

LY: conceptualization, data curation, formal analysis, investigation, writing—review and editing. QF: formal analysis, investigation, writing—review and editing. XZ: data calculation and manuscript revision. BH: conceptualization, supervision, funding acquisition, validation, investigation, visualization, methodology, project administration, and writing—original draft.

## Conflict of Interest

The authors declare that the research was conducted in the absence of any commercial or financial relationships that could be construed as a potential conflict of interest.
